# Comparison of the effects of rapid maxillary expansion and surgically assisted rapid maxillary expansion in the sagittal, vertical and transverse planes

**DOI:** 10.4317/medoral.17389

**Published:** 2011-12-06

**Authors:** Ahmet Y. Gungor, Hakan Türkkahraman, Timucin Baykul, Huseyin Alkis

**Affiliations:** 1 Assistant Professor, Department of Orthodontics, Faculty of Dentistry, University of Mustafa Kemal; 2 Associate Professor, Department of Orthodontics, Faculty of Dentistry, University of Suleyman Demirel; 3 Associate Professor, Department of Oral Surgery, Faculty of Dentistry, University of Suleyman Demirel; 4 Research Assistant, Department of Orthodontics, Faculty of Dentistry, University of Suleyman Demirel

## Abstract

Objective: The aim of this study was to evaluate and compare the effects of rapid maxillary expansion (RME) and surgically assisted RME (SARME) in the sagittal, vertical, and transverse planes. Study design: Orthodontic records of 28 patients were selected retrospectively and divided into two treatment groups. Group 1 comprised 14 patients (4 boys, 10 girls, mean age 14.2 ± 0.74 years) who had been treated with RME. Group 2 comprised 14 patients (4 boys, 10 girls, mean age 19.6 ± 2.73 years) who had been treated with SARME. Measurements were performed on lateral and posteroanterior cephalograms and dental casts obtained before (T0) and after (T1) expansion. Results: Statistically significant differences were found in soft tissue convexity angle, anterior face height, and upper nasal width in group 1, and in U1–NA length and posterior face height measurements in group 2 (P<.05). In both groups significant increases were found in interpremolar, intermolar, maxillary, and lower nasal widths and in anterior lower face height (P<.01). Statistically significant intergroup differences were found in the ANB angle (P<.05) and maxillary intercanine (P<.01) measurements. Conclusion: With both RME and SARME, successful expansion of maxillary dentoalveolar structures and nasal cavity and palatal widening were achieved. Sagittal plane effects of SARME were similar to those of RME on dental skeletal and airway measurements.

** Key words:** Surgically assisted rapid maxillary expansion, Rapid maxillary expansion, Airway, Transverse deficiency.

## Introduction 

Rapid maxillary expansion (RME) is a common treatment modality for younger patients for correction of maxillary transversal deﬁciency (1). The goal of the treatment is to widen the midpalatal suture by applying a laterally directed force against the teeth and marginal alveolar bone (2). RME can be used successfully in children and adolescents before sutural closure (1), but in non-growing adolescents and young adults, success rate of maxillary expansion decreases as sutures close (1). Maturation level of the patient is an important factor when considering the effects of RME on craniofacial structures, and RME treatment has been found more effective in children than in adults (3). Although it may be possible to achieve maxillary expansion in older patients, the results are neither as predictable nor as stable (4). At this point, surgically assisted RME (SARME) is the alternative for adolescents, and in adults, SARME is the only option for widening the maxilla; however, complications of the surgical procedure (5) and financial cost limit the applicability of the treatment to all adult patients. 

In the literature, a vast number of studies have evaluated the effects of RME on craniofacial structures (6-12). Generally, only the effects of RME on transverse plane have been evaluated since the major differences and treatment goals focus on this plane (8,10,13,14); however, the effects of a treatment modality should be evaluated in all planes of the cranium. Significant differences also occur in sagittal and vertical planes after RME procedure, but many studies omit these changes (8,10,13,14). In the literature, studies comparing RME and SARME are relatively few (1,8,15-18), and to our knowledge, none of them has compared their effects on dental, skeletal, and airway structures in sagittal, vertical, and transverse planes. 

Therefore, the aim of this study was to evaluate and compare the effects of RME and SARME in the sagittal, vertical, and transverse planes. The null hypothesis to be tested states that no difference exists between the effects of the two treatment methods in three planes.

## Material and Methods

Orthodontic records of 28 patients were selected retrospectively from the archives of Suleyman Demirel University, Faculty of Dentistry, Department of Orthodontics. Selection criteria included no previous orthodontic treatment, previous treatment with RME or SARME, no additional fixed appliances during expansion, and acceptable cooperation. Selected patients were divided into two treatment groups. Group 1 comprised 14 patients (4 boys, 10 girls, mean age 14.2 ± 0.74 years) who had been treated with RME. Group 2 comprised 14 patients (4 boys, 10 girls, mean age 19.6 ± 2.73 years) who had been treated with SARME. All patients had been treated under the supervision of the same clinician (H. T.). Bonded type Hyrax expanders with occlusal-coverage had been used in group 1, while banded type Hyrax expanders had been used in group 2. In group 1, screws were activated two turns per day (0.5 mm/d). In group 2, the screws were activated 1 mm just after the surgical interventions, which were carried out under local anesthesia. Standard horizontal osteotomy and midpalatal suture separation were performed, but the pterygoid plates were not separated from the maxilla. After a 7-day latent period, screws were activated two turns per day (0.5 mm/d). In both groups, the screws were activated until the necessary amount of expansion was achieved. After completion of the activation, screws were fixed with ligature wires and light cure band adhesives and left in place for a 4-month retention period.

**Figure 1:**
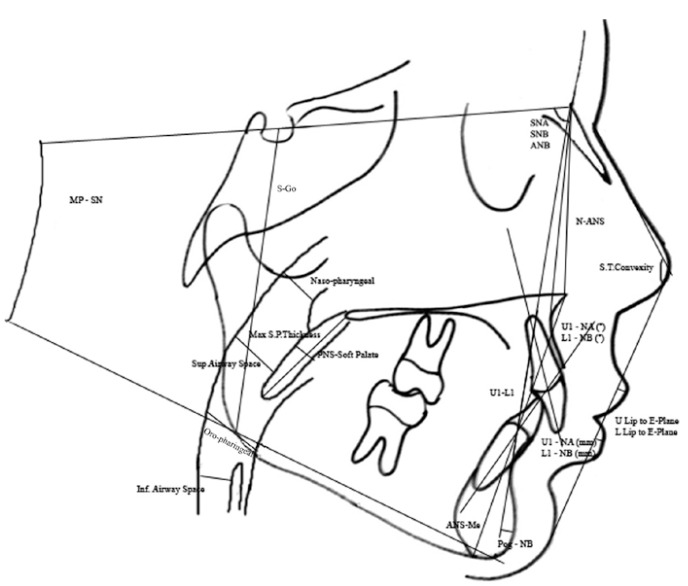
Measurements on lateral cephalometric films.

**Figure 2:**
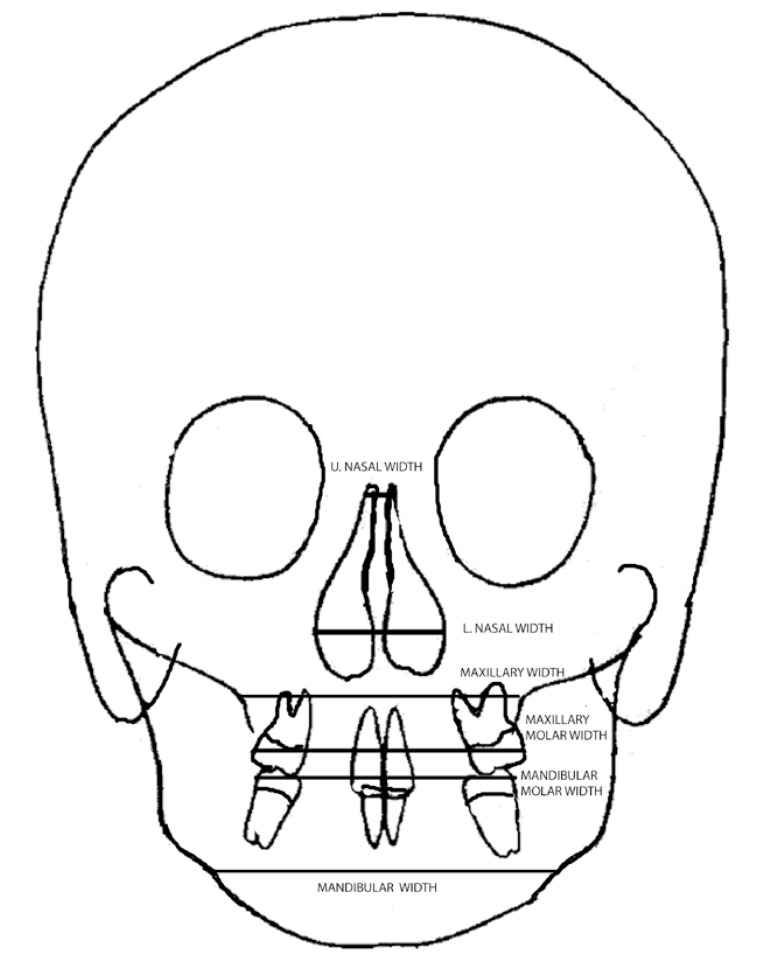
Measurements on posteroanterior cephalometric films.

A total of 38 measurements were performed on lateral (Fig. 1) and posteroanterior cephalograms (Fig. 2), and dental casts were obtained before (T0) and after (T1) expansion. 

Cephalometric landmarks were marked and digitized by one author (A.Y.G.) and measured using Dolphin imaging software version 10.5 (Dolphin Imaging & Management Solutions, Chatsworth, USA). Intercanine, interpremolar, and intermolar distances were measured from casps of the teeth. Dental cast measurements were performed with a digital caliper (Guilin Measuring and Cutting Tool Works, Guilin, China).

## Statistical method

All measurements of 10 subjects were repeated two weeks later to determine the measurement error. The Bland Altman test was used to check for differences between two sets of measurements. Descriptive statistics were calculated for all measurements, and the Wilcoxon sign test was used to evaluate treatment-induced changes within each group. The Mann-Whitney U was used for intergroup comparison. Significance for all statistical tests was predetermined at P <.05. All statistical analyses were conducted using SPSS version 11.0.0 (SPSS Inc., Chicago, IL, USA).

## Results

The results of the descriptive statistics and intragroup comparisons of cephalometric variables are presented in  Table 1 and  Table 2.  Table 3 and  Table 4 show intergroup comparisons of measurements at T0 and T1. The Bland Altman tests showed intra-examiner agreement for all cephalometric variables.

 -Intragroup changes

In group 1, among the sagittal plane measurements, only soft tissue convexity angle decreased significantly (P< .05). In the vertical plane, significant increases in anterior face height (P< .05) and in lower face height (P< .01) were found. In the transverse plane, upper (P< .05) and lower (P< .01) nasal widths (P< .01), maxillary width (P< .01), maxillary molar width (P< .01), maxillary intercanine (P< .01), interpremolar and intermolar widths (P< .01), and mandibular interpremolar widths (P< .05) increased significantly. 

In group 2, among sagittal plane measurements, significant differences were found only in U1–NA (mm) (P< .05). In the vertical plane, posterior and lower face heights increased significantly (P< .05). Most of the significant differences were found in transverse plane measurements. Lower nasal width, maxillary width, maxillary molar width, maxillary intercanine width, maxillary interpremolar widths, maxillary intermolar width, and mandibular intermolar width increased significantly (P< .01).

 -Intergroup comparison

At the start of treatment, most measurements were comparable in both groups. In group I, patients had a smaller ANB angle (P <.05), more retrusive upper and lower lips (P <.05), wider upper airway space (P <.05), longer lower face height but shorter anterior face height (P <.05), wider maxillary width (P <.05), but narrower mandibular molar width (P <.05). 

At the end of treatment, patients in group I had more retrusive upper lips (P <.05), longer lower face height but shorter anterior face height (P <.05), wider maxillary width (P <.05) but narrower mandibular molar width (P <.05) and maxillary intercanine width (P <.01).

## Discussion

The aim of this study was to evaluate and compare the effects of RME and SARME in sagittal, vertical, and transverse planes. To increase the comparability of the two treatment methods and minimize the contribution of growth and development, the oldest cases who received RME treatment were selected from the archives. Non-homogenous ages of the groups, is the limitation of the study. Indications for SARME include any case where orthodontic maxillary expansion has failed; therefore, older patients were treated with SARME than RME. Thus, results should be considered in terms of this limit. 

With both methods, no statistically significant differences were found in positions of maxillary and mandibular base relative to each other and to the cranial base. Before treatment, a smaller ANB angle was found in group I, whereas after treatment both groups were similar. This result may be explained by continuing forward growth of the maxilla in group I. Similar to our results; Altug Atac et al. (1) found statistically significant forward displacement of the maxilla only in an RME group. They explained the forward displacement by occlusal coverage of the expanders. They suggested that occlusal coverage of the expanders helped to unlock the occlusion and set the maxilla free. But when there are a few subjects, the possibility of type II error should have been considered in all studies. Many authors have reported that the maxilla moves forward with the use of RME appliances because of the rotational opening (19-25).

**Table 1:**
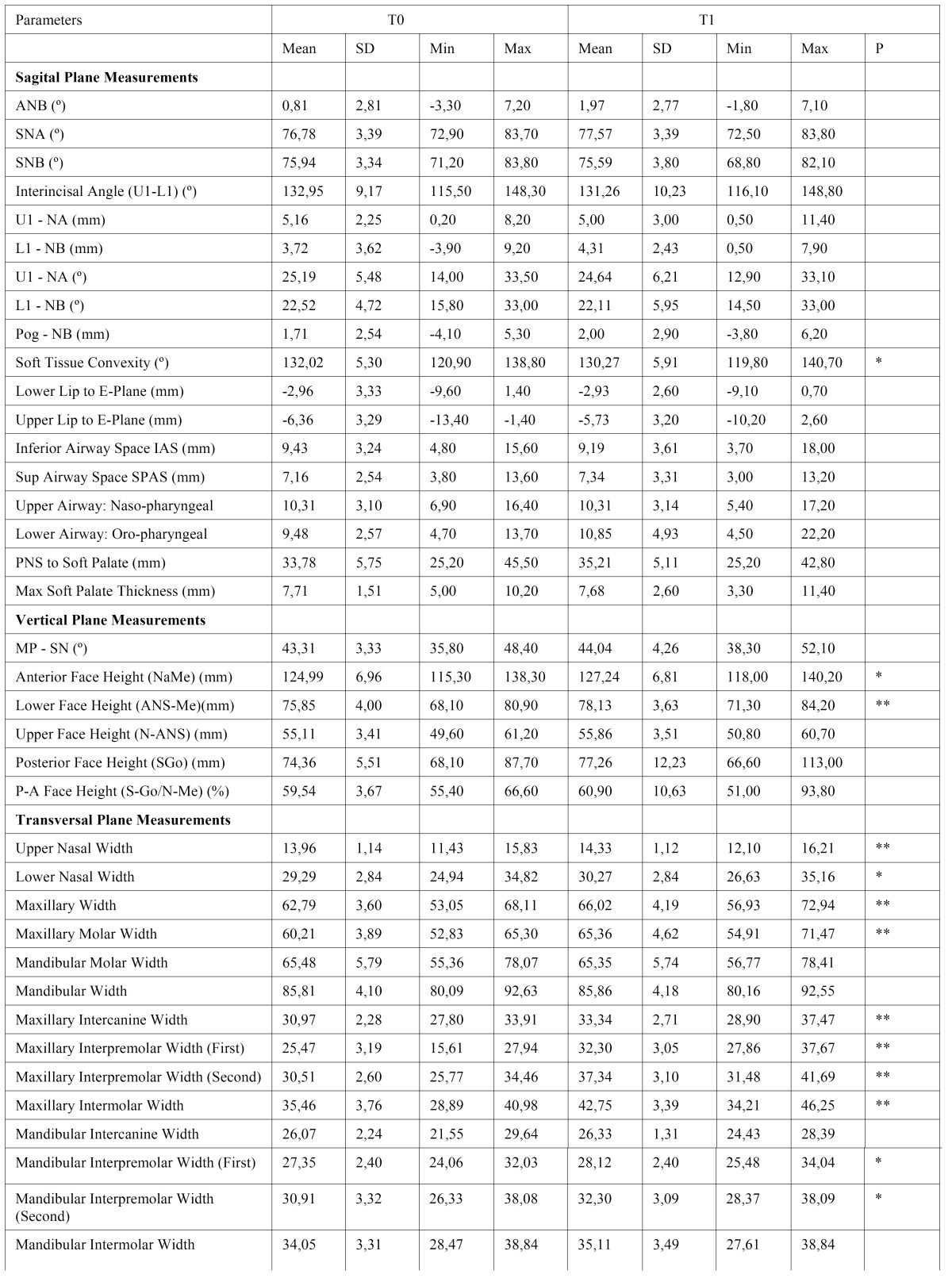
Treatment induced changes in the group I (Wilcoxon test, *p<.05, **p.<.01).

**Table 2:**
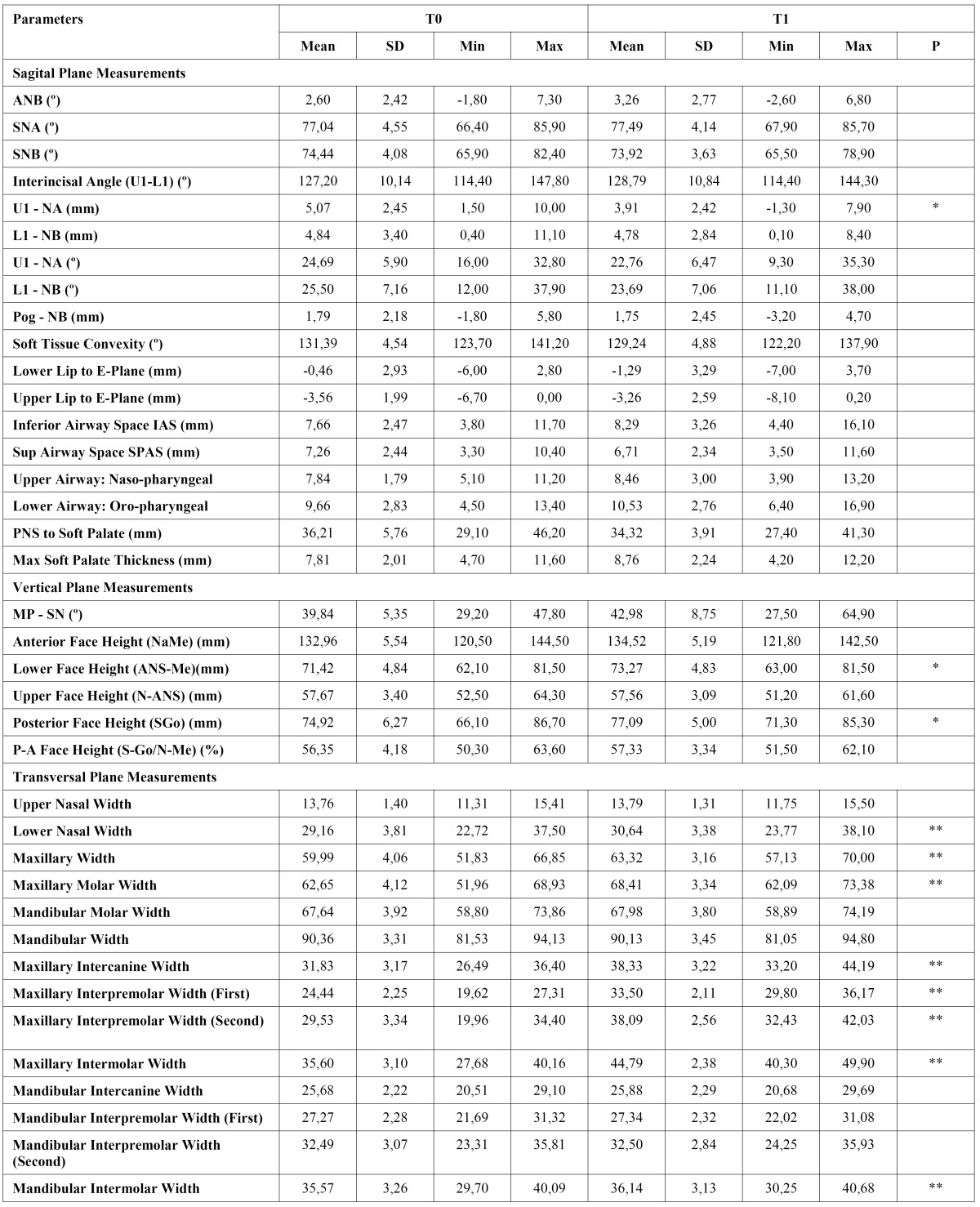
Treatment induced changes in the group II (Wilcoxon test, *p<.05, **p.<.01).

**Table 3:**
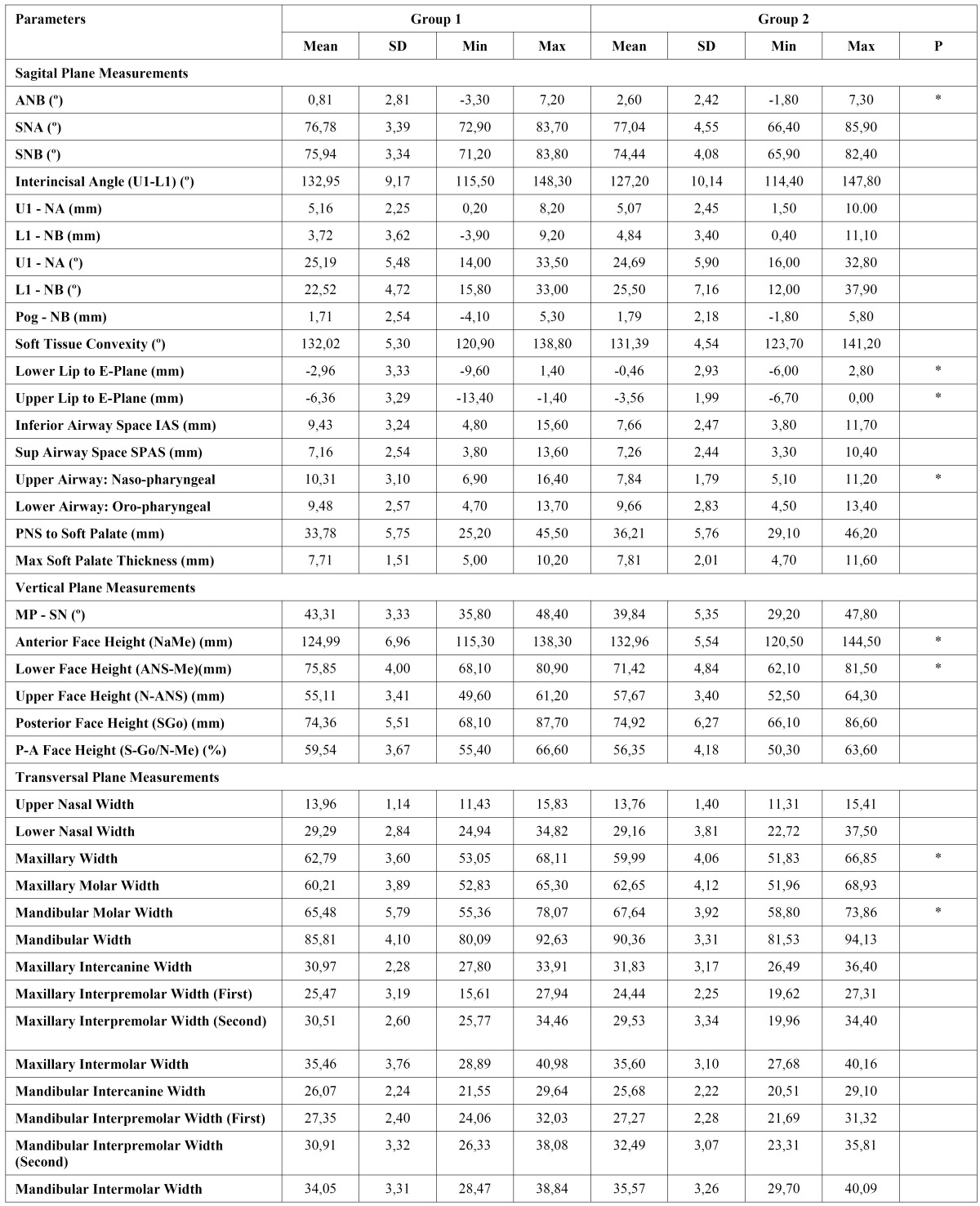
Statistical comparison of the cephalometric variables at the beginning of the observation period (Mann-Whitney U test; *p<.05, **p.<.01).

**Table 4:**
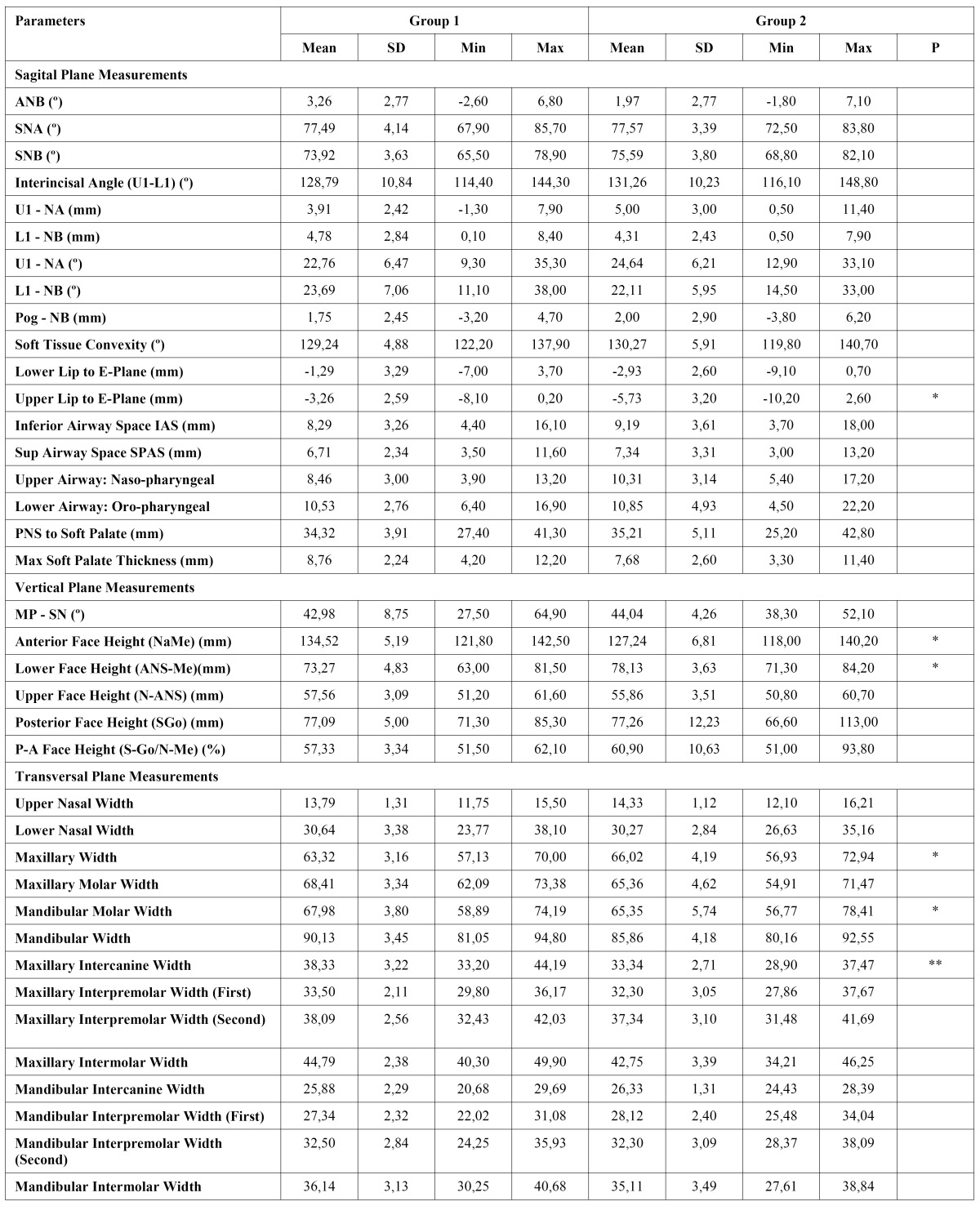
Statistical comparison of the cephalometric variables at the end of the observation period (Mann-Whitney U test; *p<.05, **p.<.01).

Altug Atac et al. (1) observed forward displacement of the mandible in the RME group, although a downward and backward displacement of the mandible was observed in other studies (6,23-25). In our study, we found no difference in mandibular position before and after treatment in both groups. Similarly Garib et al. (6) suggested that RME did not affect mandibular growth. 

Retrusion of upper incisors has been reported in various studies on RME and SARME (1,26,27). In this study we found significant retrusion of upper incisors in the SARME group. On the other hand, the RME group showed no significant differences in upper and lower incisor measurements. Retrusion of upper incisors in the SARME group could be explained by stretching of interceptal fibrils between left and right first incisors during expansion. After expansion, while interceptal fibrils close the space between left and right first incisors, retrusion of the upper incisors occurs. Since this condition is the same for two groups it seems to be more effective in SARME group. 

In both groups, significant increases were found in lower face heights. This result is in accordance with other studies for RME (1,20,25,28) and for SARME (1). Altug Atac et al. (1) found an anterior rotation of maxillary dimension in the RME group. They explained this process by resistance of the sutures in the RME group which were released in SARME group. Increases in anterior face heights could be explained by premature contacts of posterior teeth caused by a triangular widening pattern of the maxilla. 

Although increases in posterior face height were determined in both groups, only the increase in group 2 reached a statistically significant level. There were also no significant differences in posteroanterior face height proportion before and after treatment in either group, revealing that posterior and anterior face heights could be effected similarly. 

Significant transverse increases were observed in nasal cavity, maxillary base, and maxillary dentoalveolar structures in both groups. In the SARME group we achieved 5.15 (± 0.43) mm of expansion of the maxillary molar width and 3.23 (± 0.55) mm of expansion of the maxillary base. Similarly, 5.76 (± 0.44) and 3.33 (± 0.60) mm of expansion of maxillary molar width and maxillary base were obtained by RME, respectively. In both groups the greatest widening occurred in the dentoalveolar area, and the widening effect of the appliances decreased through the upper structures in a triangular pattern, as reported in previous RME studies (3,22). Since no significant differences were found between groups in amount of expansion of the maxilla, indication for RME or SARME should be based on the skeletal age of the patient and maturation of the midpalatal suture. On posteroanterior cephalograms, increases in mandibular molar width have been observed after both RME and SARME treatments in previous studies (1,29); however, we found no significant differences in mandibular molar widths in either group. According to Gryson (29), bonded expansion appliances with occlusal coverage facilitate uprighting of the mandibular posterior teeth. The increase in mandibular molar widths cannot be explained only by the occlusal coverage of the appliances, because one of the groups (RME) used appliances with occlusal coverage, but the other used banded expansion appliances (SARME), and similar changes occurred in both groups. In the literature, conflicting results are reported on the effects of maxillary expansion on dimensions of the nasal airway. In the anterior region, the dimensions are evaluated in posteroanterior cephalograms with nasal cavity widths, and in the posterior region, they are evaluated in lateral cephalograms with pharyngeal airway dimensions. In this study, we found statistically significant increases in lower nasal cavity widths in both groups; however, a significant increase in upper nasal width was found only in the RME group. These differential, method-dependent effects on nasal cavity widths may be attributed to the surgical intervention performed on group 2. The amount and localization of corticotomies could affect interactions between maxillary and nasal bones. In this study, we found some differences in pharyngeal airway dimensions, but they did not reach a statistically significant level. In accordance with our results, Malkoç et al. (30) concluded that RME and SARME do not significantly affect pharyngeal airway dimensions. 

In dental cast analysis, we found significant increases in all maxillary interdental width measurements in both groups. The amount of maxillary intercanine width expansion with SARME was nearly twice that with RME. This differential result may be attributed to surgical intervention. Anteriorly, the maxilla was separated by malleting a thin osteotome between the central incisors at a level below the anterior nasal spine (1). This procedure could have facilitated expansion at the level of maxillary canine teeth. No significant differences were found in mandibular interdental measurements between groups. In the mandible, the two techniques differ in localization of the expansion. A significant increase in interpremolar width was found with RME, whereas with SARME, intermolar width increased. A finding of increased mandibular intermolar width is in accordance with Gryson’s (29) results. 

## Conclusions

• Significant differences exist between the effects of two treatment methods; thus, the null hypothesis is rejected.

• With both SARME and RME, successful expansion of maxillary dentoalveoler structures and the nasal cavity and palatal widening were achieved.

• Since no significant differences were found between the two groups in amount of expansion of the maxilla, the indication for RME or SARME should be based on the skeletal age of the patient and maturation of the midpalatal suture.
